# Autologous Fat Grafting: an Emerging Treatment Option for Complex Anal Fistulas

**DOI:** 10.1007/s11605-023-05719-4

**Published:** 2023-06-02

**Authors:** Estella Y. Huang, Beiqun Zhao, Jason Llaneras, Shanglei Liu, Sarah B. Stringfield, Benjamin Abbadessa, Nicole E. Lopez, Sonia L. Ramamoorthy, Lisa A. Parry, Amanda A. Gosman, Marek Dobke, Samuel Eisenstein

**Affiliations:** 1 Division of Colon and Rectal Surgery, Department of Surgery, University of California, San Diego, CA USA; 2 Division of Plastic Surgery, Department of Surgery, University of California, San Diego, CA USA; 3 Division of Colon and Rectal Surgery, Department of Surgery, Baylor University Medical Center, Dallas, TX USA

**Keywords:** Anorectal, Fistula, Autologous fat grafting

## Abstract

**Background:**

Autologous fat grafting (AFG) has shown promise in the treatment of complex wounds, with trials reporting good healing rates and safety profile. We aim to investigate the role of AFG in managing complex anorectal fistulas.

**Methods:**

This was a retrospective review of a prospectively maintained IRB-approved database. We examined the rates of symptom improvement, clinical closure of fistula tracts, recurrence, complications, and worsening fecal incontinence. Perianal disease activity index (PDAI) was obtained for patients undergoing combination of AFG and fistula plug treatment.

**Results:**

In total, 52 unique patients underwent 81 procedures, of which Crohn’s was present in 34 (65.4%) patients. The majority of patients previously underwent more common treatments such as endorectal advancement flap or ligation of intersphincteric fistula tract. Fat-harvesting sites and processing technique were selected by the plastic surgeons based on availability of trunk fat deposits. When analyzing patients by their last procedure, 41 (80.4%) experienced symptom improvement, and 29 (64.4%) experienced clinical closure of all fistula tracts. Recurrence rate was 40.4%, and complication rate was 15.4% (7 postoperative abscesses requiring I&D and 1 bleeding episode ligated at bedside). The abdomen was the most common site of lipoaspirate harvest at 63%, but extremities were occasionally used. There were no statistically significant differences in outcomes when comparing single graft treatment to multiple treatments, Crohn’s and non-Crohn’s, different methods of fat preparation, and diversion.

**Conclusion:**

AFG is a versatile procedure that can be done in conjunction with other therapies and does not interfere with future treatments if recurrence occurs. It is a promising and affordable method to safely address complex fistulas.

## Introduction

Anal fistulas are a common malady, with 20,000 to 25,000 new cases in the USA annually.^[1]^ The risk of fistula development after perianal abscess drainage is thought to be up to 35–54%.^[2,3]^ Patients with Crohn’s disease (CD) are thought to have a lifetime risk of 12–28% for anal fistula development and represent 1.5% of perianal fistulas.^[4]^ While some heal with medical management alone, many recur.^[5]^ Up to 90% of Crohn’s patients with fistulas require surgery, with many needing multiple procedures.^[6]^ The chronic inflammatory nature of CD increases risk of nonhealing and limits surgical options, often leading to poor quality of life.^[7]^

Current treatments attempt to target multiple pathophysiological aspects including infection, epithelialization, inflammation, cellular senescence, and nonhealing.^[8, 10]^ A combination of antibiotics, biologics, immunosuppressive agents, and surgery is typically utilized in CD fistulas.^[11]^ Despite advances, there are high rates of nonhealing and recurrence. There is still a need for a modality with consistently high long-term healing and closure rates and minimal incontinence risk, especially in the setting of CD.

Autologous fat grafting (AFG) has been shown to improve the healing potential of complex wounds including chronic scars, burns, and ulcers ^[40]^. Adipose-derived stem cells (ASCs) are a component of lipoaspirate and have shown promise in treating anal fistulas, with previous trials reporting good healing rates and safety profile.^[12,13]^ We have been treating complex anal fistulas with autologous fat grafts since 2013. We hypothesize that outcomes with our technique are similar to those seen in the trials using more expensive lab-expanded stem cell therapies with a similar safety profile.

## Materials and Methods

Using a prospectively maintained, IRB-approved database, we performed a retrospective review of patients undergoing AFG treatment for anal fistula at a single academic institution from December 2016 to November 2021. Data gathered included demographics, procedural history, medications, operative details, and clinical outcomes. Outcomes included rates of symptom improvement, clinical or radiologic closure, recurrence, complications, and worsening fecal incontinence. Patients undergoing a planned staged closure were excluded from fistula closure rate analysis. Patients with a stoma were excluded from fecal incontinence rate. Perianal disease activity index (PDAI) scores were obtained for patients who underwent combination AFG and fistula plug treatment as part of a clinical trial (IIT-2017–10,082 treatment of perianal disease using adipose-derived stem cells). Pre-procedure PDAI was obtained within 8 weeks of surgery and post-procedure PDAI obtained at 3-month follow-up. Clinical improvement was defined as decrease in pain and drainage having significant impact on patient quality of life. Clinical closure was defined as resolution of all fistula drainage and local sepsis including no perianal pain, no seton, and visual closure of the external orifice on perianal exam. Recurrence was defined as persistence of the fistula 3 months after intervention or significant enough fistula symptoms prior to the 3-month end point to require a seton placement.

Subgroup analyses were performed examining differences in outcomes based on number of procedures, IBD status, fat preparation method, diversion, and internal orifice management. All analyses were performed in R (version 4.1.2, Vienna, Austria). Wilcoxon rank-sum test was used for continuous variables and Fisher’s exact test for categorical variables. For patients with multiple AFG procedures, data from their last procedure was used in the analysis. One patient had missing recurrence and closure data and was omitted from analysis. Two-sided *p* < 0.05 was considered significant.

### Patient Selection

Both CD and non-CD fistulas were selected, often after failing more standardized treatments such as endorectal advancement flap or ligation of intersphincteric fistula tract (LIFT), though not required if not an appropriate option for the patient. Local sepsis was controlled, and if necessary, seton placement was performed 6–12 weeks prior to AFG.

### Lipoaspirate Harvest

Fat-harvesting sites and processing technique were determined by the plastic surgeons based on truncal fat availability. If applicable, stomas were sealed with an occlusive dressing prior to fat harvest. Patients with a BMI of 28 and below with a sparsity of fat deposits frequently required fat harvest from more than one donor site. Subcutaneous fat was harvested from the anterior abdominal wall, flanks, or hips under general anesthesia. The selected area was pretreated with tumescent infiltration (1L LR, 1 mL of 1:1000 epinephrine, and 50 mL of 1% lidocaine). The amount infused was estimated by doubling expected lipoaspirate volume. Ten minutes after tumescent infiltration, manual liposuction harvest was completed using a 3 mm diameter liposuction cannula (Tulip Medical, San Diego, CA).

### Lipoaspirate Processing

The decision to process the procured lipoaspirate was based on plastic surgeon preference. Unprocessed lipoaspirate consisted of simple gravity separation for around 20 min, forming an upper fatty tissue layer and a lower tumescent layer. The upper layer was collected and placed in 10-mL syringes with 12G to 14G Coleman fat transfer cannulas (Tulip Medical, San Diego, CA).

Lipoaspirate processing was performed with the REVOLVE System (REVOLVE, LifeCell Corporation, Branchburg, NJ), an adipose-processing device that filters and washes fat. The lipoaspirate was collected directly into the REVOLVE and washed with 1L LR while simultaneously being centrifuged and filtered and then transferred into 10-mL syringes with 12G to 14G Coleman fat transfer cannulas.

### AFG Protocol

Patients were placed in high lithotomy position. Local anesthetic was minimized due to its described graft toxicity. If used, it was applied superficially and peripherally to surrounding external skin or as a pudendal nerve block for anterior fistulas. Selective skin preparation was performed with chlorhexidine. Alcohol, iodine, and peroxide were avoided due to cytotoxicity.

Once the lipoaspirate was aliquoted, the fistula tract was identified via fistula probe, aggressively debrided down to healthy bleeding tissue, irrigated with sterile saline, and the external opening widened to allow for postoperative drainage. The internal opening was closed using full-thickness, 3–0 or 2–0, absorbable sutures. Using a large bore or Coleman needle, at least 4–6 mL of graft was injected around the internal orifice along with several subsequent 1 mL injections along the soft tissue surrounding the tract. Care was taken to avoid too large of a bolus injection (i.e., greater than 1 mL) as this can lead to abscess formation. Postoperatively, the patient was instructed to resume previous wound care and prescribed a 1-week course of oral antibiotics and stool softeners if their stool was formed at baseline. This protocol was modified over time, and thus, postoperative antibiotics were standard protocol only for the latter patients. Antibiotics were added because they were believed to reduce postoperative drainage, thereby protecting the mechanical portion of the repair.

Patients with numerous fistula orifices may also have their surgeries performed as a planned staged approach, where some of the fistulas were addressed and some setons were left in place. Similarly, those with particularly large internal orifices may also have had a planned staged approach where the seton was left in place and there was extensive bulking and some suture ligation performed around the internal orifice to help minimize orifice size prior to attempts at definitive treatment. Staging is highly variable and based on many patient factors, but the second procedure is generally considered at around 3 months after the initial operation, depending on how symptomatic the patient is. This may often be preceded by seton placement if it is felt that perianal sepsis is not controlled. If that is the case, repeat procedures are done 4 to 8 weeks after seton placement. This is an outpatient procedure, and patients are told to do no heavy lifting for 1 month given the sutures. Patients were generally assessed 4 weeks and 12 weeks after their surgery with those experiencing recurrences coming back for ongoing follow-up and intervention.

## Results

### Patient Demographics

A total of 81 procedures were performed on 52 unique patients. Average age was 39.6 ± 11.9, 57.7% were female, and average BMI was 26.5 ± 5.6. CD was present in 34 (65.4%) patients. When looking at all procedures, most had prior interventions, most commonly seton placement (81.5%) and LIFT or flap (29.6%). Most did not have proctitis, but among those who did, 9 (11.1%) were mild, 1 (1.2%) severe, 7 (8.6%) pouch-associated, and 2 (2.5%) diversion-associated (Table 1). Most CD patients were receiving biologic or immunomodulator treatment. The abdomen was the most common site of fat harvest at 63% (Table 2). Average follow-up length was 17.9 months (range 1–59).

**Table 1:** Demographics

	All patients (*n* = 52)	Single procedure (*n* = 36)	Multiple procedures (*n* = 16)	*p* value
Age, years (SD)	39.6 (11.9)	40.2 (13.3)	38.1 (8.2)	0.90
Female, *n* (%)	30 (57.7%)	20 (57.1%)	10 (62.5%)	0.76
Race, *n* (%)				
Caucasian	35 (67.3%)	25 (71.4%)	10 (62.5%)	
Hispanic	8 (15.4%)	5 (13.9%)	3 (18.5%)	
Black	1 (1.9%)	1 (2.8%)	0	
Asian	1 (1.9%)	1 (2.8%)	0	
Other/unspecified	7 (13.5%)	4 (11.1%)	3 (18.5%)	
BMI, kg/m^2^ (SD)	26.5 (5.6)	27.3 (6.0)	24.6 (3.9)	0.22
CD, *n* (%)	34 (65.4%)	24 (66.7%)	10 (62.5%)	0.76
Median follow-up, months (range)	12.0 (1–59)	10 (1–59)	14 (2–53)	
Prior interventions				
Seton	66 (81.5%)	29 (80.6%)	15 (93.8%)	
LIFT or flap	24 (29.6%)	11 (30.6%)	4 (25%)	
Stem cell	11 (13.6%)	0	6 (37.5%)	
Other	7 (8.6%)	5 (13.9%)	0	
None	2 (2.5%)	0	0	
Median # prior interventions (range)	3 (0–19)	2 (0–8)	4 (2–19)	
Proctitis				
Mild	9 (11.1%)	8 (22.2%)	0	
Severe	1 (1.2%)	1 (2.8%)	0	
Pouch	7 (8.6%)	0	2 (12.5%)	
Diversion	2 (2.5%)	0	0	
None	62 (76.5%)	27 (75.0%)	14 (87.5%)	
Fistula type				
Anorectal	45 (55.6%)	21 (58.3%)	10 (62.5%)	
Pouch–anal	11 (13.6%)	5 (16.7%)	1 (6.3%)	
Pouch–vaginal	12 (14.8%)	2 (5.6%)	3 (18.8%)	
Rectovaginal	11 (13.6%)	5 (13.9%)	2 (12.5%)	
Other	2 (2.5%)	2 (5.6%)	0

**Table 2:** Medications and operative details (by procedure)

Antibiotics pre-surgery	
Ciprofloxacin	10 (12.3%)
Augmentin	7 (8.6%)
Other	3 (3.7%)
None	61 (75.3%)
Antibiotics post-surgery	
Ciprofloxacin	11 (13.6%)
Augmentin	8 (9.9%)
Other	4 (4.9%)
None	58 (71.6%)
Biologics (CD only)	
Ustekinumab	29 (35.8%)
Infliximab	10 (12.3%)
Adalimumab	8 (9.9%)
Vedolizumab	4 (4.9%)
Other	2 (2.5%)
None	3 (3.7%)
Immunomodulators (CD only)	
Azathioprine	18 (22.2%)
Methotrexate	10 (12.3%)
Mercaptopurine	1 (1.2%)
None	27 (33.3%)
Donor site	
Abdomen	51 (63%)
Abdomen + other	10 (12.3%)
Extremity	15 (18.5%)
Other	5 (6.2%)
Intervention	
Stem cell	55 (67.9%)
Stem cell + plug	24 (29.6%)
Stem cell + other	2 (2.5%)

### Outcomes

**Fig. 1:**
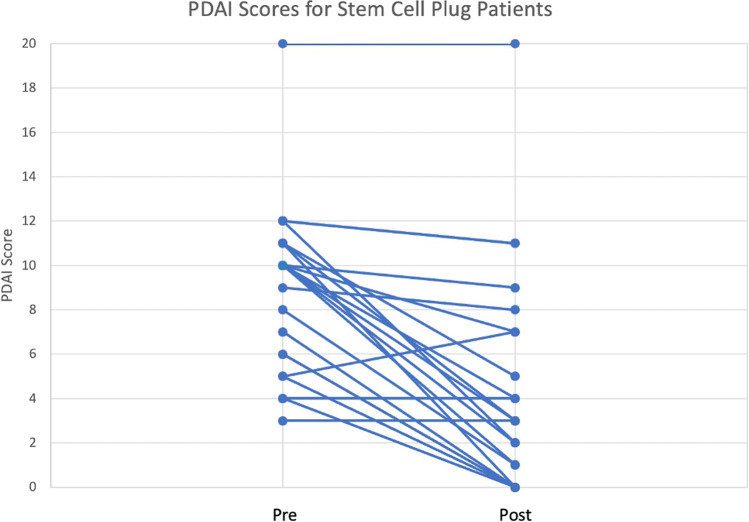
Pre- and post-PDAI scores for patients who received combination of autologous fat grafting and fistula plug treatment

When looking at all patients by last procedure, 41 (80.4%) experienced symptom improvement, and 29 (64.4%) experienced clinical closure of all tracts. The recurrence rate was 40.4%. Eight (15.4%) patients experienced complications — 7 postoperative abscesses requiring incision and drainage and 1 bleeding episode requiring bedside ligation. Excluding patients with a stoma, 1 (2.1%) had worsening fecal incontinence post-operation (Table 3). For the 23 patients who received combination of AFG and fistula plug treatment, average pre-intervention PDAI was 8.4, and average post-intervention PDAI was 2.4 (*p* < 0.0001) (Fig. 1).

**Table 3:** Outcomes in single vs multiple procedures

	All patients (*n* = 52)	Single procedure (*n* = 36)	Multiple procedures (*n* = 16)	*p* value
Improvement	41 (80.4%)	30 (83.3%)	11/15 (73.3%)	0.45
Closure	29 (64.4%)	22 (61.1%)	7/15 (46.7%)	0.99
Recurrence	14 (40.4%)	13 (36.1%)	8 (50.0%)	0.38
Complication	8 (15.4%)	6 (16.7%)	2 (12.5%)	0.99
Abscess	7	5	2	
Bleeding	1	1	0	
Worsening fecal incontinence	1 (2.1%)	1/33 (3.0%)	0/14	0.99

### Subgroup Analysis: Multiple Procedures

Thirty-six patients received a single AFG treatment, 16 received multiple, 10 CD, and 6 non-CD. Multiple procedure patients had comparable rates of improvement (73.3% versus 83.3%, *p* = 0.45), closure (46.7% versus 61.1%, *p* = 0.99), recurrence (50.0% versus 36.1%, *p* = 0.38), complications (12.5% versus 16.7%, *p* = 0.99), and worsening incontinence rates (0% versus 3.0%, *p* = 0.99). Of the multiple procedure patients, four were planned staged, five recurred after initial closure, and the remaining seven did not achieve closure before the next treatment (Table 3).

### Subgroup Analysis: Crohn’s Disease

There were 34 CD patients and 18 non-CD patients. While non-CD patients had higher closure rates (70.6% versus 50.0%, *p* = 0.23), this was not statistically significant. There were no significant differences between the CD and non-CD when looking at rates of clinical improvement (79.4% versus 82.4%, *p* = 0.99), recurrence (44.1% versus 33.3%, *p* = 0.56), complications (17.6% versus 11.1%, *p* = 0.70), and worsening fecal incontinence (10.0% versus 11.1%, *p* = 0.99) (Table 4).

**Table 4:** Outcomes in CD vs non-CD

	CD (*n* = 34)	Non-CD (*n* = 18)	*p* value
Improvement	27 (79.4%)	14/17 (82.4%)	0.99
Closure	17 (50.0%)	12/17 (70.6%)	0.23
Recurrence	15 (44.1%)	6 (33.3%)	0.56
Complication	6 (17.6%)	2 (11.1%)	0.70
Worsening fecal incontinence	3/30 (10.0%)	2 (11.1%)	0.99

### Subgroup Analysis: Method of Fat Preparation

The fat was filtered in 26 patients and unfiltered in 25 patients. There were no significant differences between the filtered and unfiltered groups when looking at rates of improvement (84.6% versus 75.0%, *p* = 0.49), closure (61.5% versus 50.0%, *p* = 0.57), recurrence (38.5% versus 44.0%, *p* = 0.78), complications (11.5% versus 16.0%, *p* = 0.70), and worsening fecal incontinence (0% versus 4.8%, *p* = 0.47) (Table 5).

**Table 5:** Filtered vs unfiltered fat

	Fat filtered (*n* = 26)	Fat unfiltered (*n* = 25)	*p* value
Improvement	22 (84.6%)	18/24 (75.0%)	0.49
Closure	16 (61.5%)	12/24 (50.0%)	0.57
Recurrence	10 (38.5%)	11 (44.0%)	0.78
Complication	3 (11.5%)	4 (16.0%)	0.70
Worsening fecal incontinence	0/25	1/21 (4.8%)	0.47

### Subgroup Analysis: Diversion

Of the 52 patients, 10 patients had fecal diversion. There were no significant differences between the diverted and nondiverted group in the rates of improvement (90.0% versus 78.0%, *p* = 0.66), closure (62.5% versus 64.9%, *p* = 0.99), recurrence (20.0% versus 45.2%, *p* = 0.17), complications (20.0% versus 14.3%, *p* = 0.64), and worsening fecal incontinence (0% versus 2.4%, *p* = 0.99) (Table 6).

**Table 6:** Outcomes by diversion

	Diversion (*n* = 10)	No diversion (*n* = 42)	*p* value
Improvement	9 (90.0%)	32/41 (78.0%)	0.66
Closure	5/8 (62.5%)	24/37 (64.9%)	0.99
Recurrence	2 (20.0%)	19 (45.2%)	0.17
Complication	2 (20.0%)	6 (14.3%)	0.64
Worsening fecal incontinence	0/5	1 (2.4%)	0.99

### Subgroup Analysis: Internal Orifice Management

Twenty-seven patients had primary ligation (PL) of their internal orifice, 16 received a fistula plug (FP), and 8 had no closure (NC). PL patients experienced more improvement at 92.3% compared to 75.0% of FP (*p* = 0.99) and 50.0% of NC (*p* = 0.02). PL also had less complications at 3.7% compared to 25.0% in FP (*p* = 0.99) and 37.5% in NC (*p* = 0.03). There were no significant differences between the three groups (PL, FP, NC) when looking at closure (66.7% versus 56.3% versus 75.0%, *p* = 0.65), recurrence (37.0% versus 31.3% versus 62.5%, *p* = 0.37), and worsening fecal incontinence (4.3% versus 0% versus 0%, *p* = 0.99) (Table 7).

**Table 7:** Outcomes by internal orifice management

	Primary ligation (*n* = 27)	Fistula plug (*n* = 16)	No closure (*n* = 8)	*p* value
Improvement	24/26 (92.3%)	12 (75.0%)	4 (50.0%)	0.026 NC vs FP: 0.36 NC vs PL: 0.02 FP vs PL: 0.99
Closure	16/24 (66.7%)	9 (56.3%)	3/4 (75.0%)	0.65
Recurrence	10 (37.0%)	5 (31.3%)	5 (62.5%)	0.37
Complication	1 (3.7%)	4 (25.0%)	3 (37.5%)	0.02 NC vs FP: 0.65 NC vs PL: 0.03 FP vs PL: 0.99
Worsening fecal incontinence	1 (4.3%)	0	0	0.99

## Discussion

Autologous AFG was comparable to other current treatments for complex anal fistulas. In our cohort, 80.4% had clinical improvement, 64.4% had clinical closure, and 40.4% had recurrence. CD patients had similar outcomes, with 79.4% improvement, 50.0% complete closure, and 44.1% recurrence. Symptom scores among AFG fistula plug patients improved significantly. The complication rate of 15.4% (mostly postoperative abscesses) can likely be explained by the fact that patients receiving AFG treatment have more complicated fistulas that are more prone to infection. AFG can be a viable and economical option with a favorable risk profile for a variety of anal fistula patients.

Current therapy for complex fistula combines medical and surgical modalities. Medical management consists of antibiotics and, in the setting of CD, anti-TNF agents such as infliximab or other biologics or immunosuppressants.^[14]^ Surgery also plays an important role. The initial step is control of perianal sepsis through drainage of any abscesses with possible placement of a draining seton to help optimize the fistula tract. Subsequent steps can involve an advancement flap, sealants, plugs, or a LIFT. Endorectal advancement flaps have good healing rates but can be challenging for revision surgery given scar tissue.^[15]^ Fibrin sealants and plugs have healing rates of less than 50% and may not be the best option for complex fistulas.^[16,17]^ LIFT has reasonable functional outcomes but may have limited success in the setting of CD.^[18]^ Both advancement flap and LIFT carry a risk of incontinence and should not be performed in the setting of proctitis given significant risk of nonhealing.^[19,20]^

AFG for complex wounds has become increasingly popular, showing promising outcomes in treating wounds like diabetic foot ulcers, chronic burns, radiation-induced wounds, and chronic scars. ^21,^^23^ The pathophysiology of AFG treatment is poorly understood and still a matter of investigation. Current theories suggest a multifactorial mechanism. One possible explanation is that the lipoaspirate forms a physical scaffold surrounding the wound, serving as a matrix for new cell migration and growth, neovascularization, and granulation tissue formation.^[21]^ Others credit individual components of lipoaspirate, specifically adipose-derived stem cells and adipokines. ^[24,25]^ Kim et al. demonstrated that lipoaspirate has a regenerative effect on human keratinocytes by increasing their migration, proliferation, and wound healing potential.^[26]^

Several high-quality studies examine the effect of ASCs on anal fistulas. The FATT 1 trial (2012) was a phase III, multicenter, randomized clinical trial investigating long-term success rates of ASC treatment.^[12]^ The three arms consisted of treatment with ASC only, ASC and fibrin glue, and fibrin glue only with healing, confirmed by imaging at 1 year, achieved in 57%, 52%, and 37%, respectively. An RCT performed by the same group improved upon the methods of the previous trial and found no significant differences at 1 year but at 2 years observed a 50% healing rate in the ASC plus fibrin glue group compared with 26% in the fibrin glue only group (*p* = 0.129).^[27]^ Panes et al. conducted ADMIRE-CD, another phase III trial investigating ASC treatment in CD patients, which yielded a 50% remission rate compared with 34% in the placebo group at 6 months.^[28]^ At 1 year, the remission rate in the ASC group was 56% compared with 39% in the control group.^[29]^ Other studies report healing rates of anywhere from 50 to 60%.^[30,31]^ These studies provide support for the long-term efficacy of anal fistula ASC treatment.

One major advantage of our technique is the cost savings associated with the use of lipoaspirates. Darvadstrocel, a suspension of expanded ASCs, was used in the ADMIRE-CD trial and received a regenerative medicine advance therapy (RMAT) designation from the FDA for complex perianal fistulas in adults with Crohn’s.^[32]^ The cost of one darvadstrocel treatment is around $65,000 and cannot be billed to insurance. Our procedure involving the use of lipoaspirate (which consists of ASCs) harvested from the patient’s own body, costs around $1000 (including the amount insurance bills for liposuction and fistula treatment), and has a 65% clinical closure rate, comparable to the 56% remission rate of the ADMIRE-CD trial patients. At our institution, the average cost for disposable supplies in cases utilizing non-filtered (syringe) fat acquisition and preparation is $200, while cases using REVOLVE cost over $800. These data do not include costs and amortization of reusable equipment. One added benefit is that US-based insurance companies have generally covered the CPT codes billed for this procedure, which include 46,600 (anoscopy), 46,280 (surgical treatment of anal fistula), 20,926 (tissue grafting), making the cost to those patients with health insurance minimal.

Harvested lipoaspirate preparation typically involves removal of nonviable components (e.g., blood cells, oil and any debris) and can use methods like washing, gravity, centrifugation, or filtration.^[33]^ Increased processing can decrease contamination and increase yield but can also damage the adipose tissue or negatively affect factors such as cytokine secretion.^[34]^ We investigated outcomes in the setting of both filtered and unfiltered fat and saw a trend in filtered fat having more improvement (84.6% versus 75.0%) and closure (61.5% versus 50.0%), but these were not statistically significant. In our study, the decision to process the procured lipoaspirate was based on plastic surgeon preference and was either done via gravity separation or the use of a REVOLVE System, an adipose-processing device that filters and washes fat. Various small studies show that different fat processing techniques lead to varying physical and biological characteristics of the grafts. Filtrated grafts tend to contain a higher percentage of fat tissue and stromal cell fraction whereas unfiltered grafts tend to contain higher volume yield. However, there have been no in vivo studies to date that demonstrate clinical differences between the two fat processing methods.^[35]^ Nevertheless, in both methods, the use of autologous fat grafts was easily accessible, safe, not immunogenic, and could be used at time of surgery.^[13,36]^ More investigation is needed to determine clinical differences between methods of lipoaspirate preparation to establish a standardized protocol.

Anal fistulas can be especially disabling and morbid in the setting of CD.^[37]^ While our CD patients experienced decreased success rates compared with non-CD patients, they still had 79% improvement and 50% closure, with 44% recurrence, superior comparable to the 44% recurrence rate of infliximab and non-cutting seton placement.^[38]^ This is also comparable to reported closure rates of around 58% in endorectal advancement flap procedures and 48% for LIFT procedures in Crohn’s patients.^[39,40]^ This shows that AFG can be a good alternative to other surgical treatment, given that it is less invasive with equivalent success rates.

The treatment of complex fistulas often requires multiple attempts. Not all treatments can be easily performed more than once given that some create barriers for future procedures. For example, creating an endorectal advancement flap or performing a LIFT can lead to scar tissue, making subsequent repair attempts more difficult. Conversely, AFG can be done multiple times and does not preclude the possibility of other procedures in the event of a recurrence. In our series, the average patient underwent 1.6 AFG procedures. We compared the outcomes of single to multiple procedure patients and found similar improvement, closure, and complication rates, showing that multiple procedures for the same patient was just as efficacious. This may also demonstrate that AFG can create a more favorable milieu for subsequent treatments.

Our study was retrospective and single-center. Our dataset is very heterogenous, and surgical technique has evolved over time, making it difficult to isolate variables when performing analyses. Given the size of the series, it was difficult to perform a rigorous multivariable analysis, and some analyses may be underpowered. The single-center nature may limit generalizability. Larger, prospective, long-term studies are needed to further investigate subsets of patients this treatment is optimal for. Patients with positive outcomes generally did not require post-procedural MRIs, making the assessment of MRI resolution challenging for this population. Generally, only fistulas failing treatment went on to get further MRIs, making the radiologically assessed closure rate artificially low. A significant argument can be made that radiologic assessment is not necessary if the patient’s fistula is asymptomatic, as the patient’s ultimate goal is clinical resolution.

## Conclusion

Anal fistulas are challenging, and it is helpful to have a variety of options when tailoring therapy for patients, but there is yet to be a dependable treatment with consistently high, long-term success rates. Autologous fat grafting is a versatile, safe procedure that can be repeated several times and performed in conjunction with other therapies. It has emerged as a promising method to safely address complex fistulas in a way that is affordable for patients.
